# CD44v6 specific CAR-NK cells for targeted immunotherapy of head and neck squamous cell carcinoma

**DOI:** 10.3389/fimmu.2023.1290488

**Published:** 2023-11-10

**Authors:** Ioana Sonya Ciulean, Joe Fischer, Andrea Quaiser, Christoph Bach, Hinrich Abken, Uta Sandy Tretbar, Stephan Fricke, Ulrike Koehl, Dominik Schmiedel, Thomas Grunwald

**Affiliations:** ^1^ Fraunhofer Institute for Cell Therapy and Immunology (IZI), Leipzig, Germany; ^2^ Leibniz Institute for Immunotherapy, Division of Genetic Immunotherapy, Regensburg, Germany; ^3^ Institute for Clinical Immunology, University of Leipzig, Leipzig, Germany

**Keywords:** CAR-NK cells, head and neck cancer, CD44v6, NK cells, solid tumor, immune cell therapy, chimeric antigen receptor

## Abstract

Head and neck squamous cell carcinoma (HNSCC) is a major challenge for current therapies. CAR-T cells have shown promising results in blood cancers, however, their effectiveness against solid tumors remains a hurdle. Recently, CD44v6-directed CAR-T cells demonstrated efficacy in controlling tumor growth in multiple myeloma and solid tumors such as HNSCC, lung and ovarian adenocarcinomas. Apart from CAR-T cells, CAR-NK cells offer a safe and allogenic alternative to autologous CAR-T cell therapy. In this paper, we investigated the capacity of CAR-NK cells redirected against CD44v6 to execute cytotoxicity against HNSCC. Anti-CD44v6 CAR-NK cells were generated from healthy donor peripheral blood-derived NK cells using gamma retroviral vectors (gRVs). The NK cell transduction was optimized by exploring virus envelope proteins derived from the baboon endogenous virus envelope (BaEV), feline leukemia virus (FeLV, termed RD114-TR) and gibbon ape leukemia virus (GaLV), respectively. BaEV pseudotyped gRVs induced the highest transduction rate compared to RD114-TR and GaLV envelopes as measured by EGFP and surface CAR expression of transduced NK cells. CAR-NK cells showed a two- to threefold increase in killing efficacy against various HNSCC cell lines compared to unmodified, cytokine-expanded primary NK cells. Anti-CD44v6 CAR-NK cells were effective in eliminating tumor cell lines with high and low CD44v6 expression levels. Overall, the improved cytotoxicity of CAR-NK cells holds promise for a therapeutic option for the treatment of HNSCC. However, further preclinical trials are necessary to test *in vivo* efficacy and safety, as well to optimize the treatment regimen of anti-CD44v6 CAR-NK cells against solid tumors.

## Introduction

Cancer immunotherapy has witnessed significant success with the emergence of chimeric antigen receptor T-cell (CAR-T) therapy, particularly in the treatment of blood cancers. FDA-approved anti-CD19 CAR-T therapies such as Kymriah^®^, Yescarta^®^, and Tecartus^®^ (1–3) demonstrated long-lasting remission effect in patients with various B-cell malignancies (4), paving the way towards CAR therapy development for other malignancies. However, the translation of CAR-T success to solid tumors is still challenging. Though currently clinically manageable (5), life-threatening side effects associated with CAR-T therapies such as cytokine release syndrome (CRS) and immune effector cell-associated neurotoxicity syndrome (ICANS) (5–7) led the search for alternative immune cell types. In this context, the use of NK cells for developing CAR-based cellular therapy options offers several advantages over T cells. First, CAR-NK cells are associated with a reduced risk of inducing graft-versus-host disease (GvHD) due to their non-MHC-restricted mechanism of target recognition (8, 9). Therefore, they can be administered as an allogeneic, off-the-shelf cell therapy product generated from healthy donor cells instead of autologous patient-individual immune cells. NK cells can be obtained from various sources including peripheral blood (10), umbilical cord blood (11, 12), induced pluripotent stem cells (13) or cell lines like NK-92 (14). Regarding their off-the-shelf use, it was previously reported that one unit of cord blood could yield approximately 100 doses of CAR-NK cells (11), potentially undercutting the $375,000 per infusion cost of CAR-T cells (15). Moreover, CAR-NK cell products are considered safer for administration because they possess a distinct cytokine profile from the proinflammatory T cell cytokines associated with CRS occurrence (16). Their safe administration was demonstrated in a clinical trial (clinical trials.gov NCT03056339) (17).

Head and neck squamous cell carcinomas (HNSCC) represent a major challenge in cancer treatment. According to GLOBCAN estimates, in 2020 a number of 377.713 new cases and 177.757 new deaths worldwide were attributed to cancers of the lip and oral cavity (18). Current treatment plans for HNSCC involve a multimodal approach, with surgery or radiation therapy being a curative option for early-stage disease (19). However, locally advanced cases carry a higher risk of recurrence and distant metastases, resulting in worse prognosis (20). The introduction of targeted immunotherapeutic options, such as cetuximab (21), an anti-EGFR monoclonal antibody, and later the inclusion of immune checkpoint inhibitors targeting PD-1, like nivolumab and pembrolizumab (22, 23), broadened treatment possibilities for patients with locally advanced HNSCC. However, a need for effective immune cell therapies remains. To date, only a few surface markers have been studied as possible targets for CAR therapy in HNSCC. These include EGFR (24, 25), HER2 (26), PD-L1 (27), MUC1 (28) and CD44v6 (29). CD44v6, an isoform of the CD44 glycoprotein, emerges as a promising target for cellular therapy in HNSCC. CD44v6 is expressed in various malignancies like multiple myeloma (30), acute myeloid leukemia (31), breast (32), gastric (33) and colorectal cancers (34), as well as head and neck cancers (35). CD44v6 is also expressed by 1.5-3.6% healthy immune cells of the myeloid lineage (36), while oral mucosa keratinocytes were found to express the CD44v1 and v2 isoforms of CD44 (37). Preclinical studies demonstrated the potential of CAR-T cell therapy targeting CD44v6 in HNSCC (29), lung and ovary adenocarcinoma (38), as well as myeloid leukemia and multiple myeloma (39). Clinical trials utilizing CD44v6 CAR-T cells have been launched for both blood cancers (clinical trials.gov NCT04097301) and solid tumors (clinical trials.gov NCT04427449), highlighting the therapeutic potential of this target. Recent research has shown the efficacy of CD44v6 CAR-NK cells against triple-negative breast cancer *in vitro* (40), while in a phase 2 clinical trial, anti-PD-L1 CAR-NK cells in combination with the anti-PD-1-antibody pembrolizumab and the IL-15 superagonist complex N-803 are used to treat recurrent or metastatic gastric or head and neck cancers (clinical trials.gov NCT04847466).

In this *in vitro* proof of principle study, we explore the use of gamma retroviral vector-generated CAR-NK cells against CD44v6 to augment the innate cytotoxicity of NK cells against HNSCC cell lines. In a side-by-side comparison, we tested the efficacy of three viral envelopes in transducing primary NK cells and checked for the impact on NK cell surface markers.

## Materials and methods

### Ethics statement

This work was performed with NK cells from anonymous healthy blood donors and was approved by the State Chamber of Physicians of Saxony, Germany, under the ethical vote number EK-BR-79/21-1.

### Cell lines and primary cells culture

HEK293-T cells (ACC 635) obtained from DSMZ (Braunschweig, Germany) were grown in DMEM GlutaMAX (Gibco, New York, United States) supplemented with 10% fetal calf serum (FCS; Gibco, New York, United States) and 1% (v/v) penicillin-streptomycin (pen./strep., Gibco, New York, United States). Prof. Reidar Grénman, University of Turku, Finland kindly provided the human head and neck cancer cell lines UT-SCC-14 (tongue squamous cell carcinoma) and UT-SCC-42B (laryngeal squamous cell carcinoma). UT-SCC-14 and UT-SCC-42B cells were grown in DMEM high glucose medium (Sigma-Aldrich, St. Louise, United States) supplemented with 10% (v/v) FCS and 1% (v/v) GlutaMax (Gibco, New York, United States). The tongue squamous cell carcinoma derived SCC-25 cell line (ACC 617) obtained from DSMZ (Braunschweig, Germany) was kept in DMEM/F12 high glucose medium (Gibco, New York, United States) supplemented with 10% (v/v) and 1% (v/v) GlutaMax. The glioblastoma cell line LN-299 purchased from ATCC (CRL-2611) was cultivated in DMEM high glucose medium (Sigma-Aldrich, St. Louise, United States) supplemented with 10% (v/v) FCS. All adherent cancer cell lines were harvested after a 20-30 min incubation with accutase cell detachment solution (Corning, New York, United States). NK-92, a malignant non-Hodgkin’s lymphoma cell line (ATCC CRL-2407) was kept in RPMI 1640 HEPES modified medium (Sigma-Aldrich, St. Louise, United States) supplemented with 20% (v/v) FCS, 1% (v/v) GlutaMax, 1% (v/v) MEM non-essential amino-acids (Sigma-Aldrich, St. Louise, United States) and 100 IU/ml recombinant rhIL-2 Proleukin^®^S (Clinigen Healthcare B.V, Schiphol, Netherlands). All cell lines were cultivated at 37°C, 5% (v/v) CO_2_, and 95% (v/v) humidity and were split every 2 to 3 days. Regular testing for Mycoplasma was performed for all cell lines using a LookOut^®^ Mycoplasma PCR Detection Kit (Sigma-Aldrich, St. Louise, United States).

Primary NK cells were purified from healthy donor buffy coats by negative selection using the RosetteSep™ human NK cell enrichment cocktail according to the manufacturer’s instructions (STEMCELL Technologies, Vancouver, Canada). After NK cell isolation, 1 × 10^6^ cells per well were cultivated in a 24-well plate, in 1 ml of NK MACS^®^ medium (Miltenyi Biotec, Bergisch-Gladbach, Germany) supplemented with 5% (v/v) human serum (Sigma-Aldrich, St. Louise, United States), 500 IU/ml of recombinant rhIL-2 Proleukin^®^S and 140 IU/ml of IL-15 (Miltenyi Biotec, Bergisch-Gladbach, Germany). NK cell purity was determined by flow cytometry post-isolation. All NK cell-isolations had a purity higher than 72% on the day of isolation and higher than 95% on the day of transduction.

### Transgene constructs

The anti-CD44v6 CAR construct was cloned in the pBullet (41) gamma retroviral vector by replacing the BW431/26-scFv-Fc-CD28-CD3ζ CAR (42). An enhanced GFP (EGFP) reporter gene flanked by the *PacI* (5´) and *XhoI* (3´) restriction sites (Eurofins, Luxembourg, Luxembourg) was first cloned into the original vector construct, downstream of the CAR construct. For the construction of the anti-CD44v6 specific CAR, the anti-CD44v6 single chain variable fragment (scFv) sequence was synthetized in a lentivirus expression plasmid from Invitrogen GeneArt (Thermo Fisher, Waltham, MA, USA). In brief, the anti-CD44v6 scFv with a VL-VH orientation consists of the following encoding sequences: CD8a signaling peptide, sequences of the VL and VH region of the humanized BIWA8 antibody (43) connected by a (G_4_S)_3_ linker. The scFv was amplified from the lentivirus expression plasmid via PCR using the following primers (Biomers.net GmbH, Ulm, Germany): BIWA_for (AGCCACCATGGCCTTACCAGTGAC) and BIWA_VL-VH_rev (GCATGGATCCAGGCTGCTCACGGTCACCAGG). PCR products were purified using a Macherey-Nagel™ NucleoSpin™ Gel and PCR Clean-up Kit according to the manufacturer’s instructions (Machery-Nagel GmbH & Co.KG, Düren, Germany). Ligation of the scFv into the pBullet gRV CAR backbone was performed with 50 ng vector and 16 ng of scFv insert at a molar insert to vector ratio of 3:1, using T4 DNA Ligase (New England Biolabs, Ipswich, UK) according to the manufacturer’s instructions and incubated overnight at 16°C. For the bacterial transformation step, 4 to 8 µl of the ligation mixture was added to 50 µl of chemical competent NEB stable *E. coli* (C3040, New England Biolabs, Ipswich, UK) and incubated on ice for 30 min. This was followed by a 30 s heat shock at 42°C and a cool down step of 2 min on ice. 950 µl of NEB^®^ 10-beta/stable Outgrowth (New England Biolabs, Ipswich, UK) medium was added and the bacteria was incubated at 30°C, 400 rpm for 1 hour (h). Next, the transformed bacteria were plated on LB agar plates containing 100 µg/ml ampicillin. Bacterial colonies were grown overnight at 30°C and clones were picked on the next day and expanded in appropriate LB medium. Plasmids were isolated from bacterial liquid cultures using the E.Z.N.A.^®^ DNA Mini Kit according to the manufacturer’s instructions (Omega Bio-tek Inc., Norcross, USA). DNA sequences were checked by Sanger sequencing (Microsynth AG, Balgach, Switzerland), the clone with the correct sequence was further expanded and plasmid-working stocks were generated using the EndoFree Plasmid Maxi Kit (10) according to the manufacturer’s instructions (Qiagen GmbH, Venlo, Netherlands).

Lentivirus vectors encoding firefly luciferase (F.luc) and puromycin (Puro) were used to generate tumor target cell lines that have a stable expression of the luciferase enzyme. The used pGreenFire_Puro_Luciferase plasmid was generated by exchanging the GFP reporter in the pGreenFire1-CMV Plasmid (TR011PA-1-SBI System Biosciences) backbone with a puromycin resistance gene.

### Gamma retroviral vector generation, concentration and titration

Transient transfection of HEK293-T cells using a calcium phosphate transfection kit (Thermo Fisher, Waltham, MA, USA) was used to generate gRV vector particles. The viral packaging system used included a plasmid expressing the Gag and Pol retroviral core proteins (pHIT60) (41) and one of the three envelope plasmids: envelope derived from the baboon endogenous virus envelope (BaEVRless, BaEV for short) (44), feline leukemia virus (FeLV, termed RD114-TR, RD114 for short) (45) and gibbon ape leukemia virus (GaLV) (41), respectively. One day before transfection, 7 × 10^6^ HEK293-T cells were seeded per flask into 10 x T175 flasks. In total, 35 μg plasmid DNA (plasmids expressing transfer gene, Gag/Pol and envelope in a ratio of 5:3:2) was mixed with 1.26 ml 2M calcium chloride and topped with sterile ultra-filtrated water up to 10.5 ml. The 2X HEPES buffered saline was added dropwise to the DNA mix for a final 1:1 dilution and incubated for 10 min at room temperature. The culture medium was exchanged to 35 ml DMEM GlutaMAX supplemented with 1.5% (v/v) FCS and 1% (v/v) pen./strep. solution before the transfection solution was added dropwise over the HEK293-T cells. Eight hours later, the medium was replaced with fresh DMEM GlutaMAX supplemented with 10% (v/v) FCS and 10.000 U/ml penicillin; 10 mg/ml streptomycin; the flasks were further incubated for approximately 72 h. Three days after transfection, the cell culture supernatant was collected, centrifuged down for 5 min, 800 x g to get rid of cell debris and then filtered through a 0.45 μm filter. Part of the viral vector suspension was kept and frozen, while the rest of the suspension was mixed at a 1:3 ratio with the Retro-X concentrator solution (Takara Bio Inc., Kusatsu, Japan) and incubated at 4°C overnight. The following day, the vector suspension was centrifuged for 45 min, at 4°C and 1,500 x g; the supernatant was discarded, and pellets were resuspended in 2-3 ml DMEM GlutaMAX medium supplemented with 10% (v/v) FCS and 1% (v/v) pen./strep. for a 75-90 x concentration effect. Concentrated viral vector stocks were aliquoted and frozen at -80°C. To determine vector titers, frozen vector aliquots were titrated on HEK293-T cells using six serial dilutions of the vector particles. HEK293-T transduction efficacy was checked by flow cytometry after 4 days and was quantified as percentage of the EGFP+ cells.

### Anti-CD44v6 CAR-NK generation

Primary NK (pNK) cell transduction was done in 24-well flat-bottom plates for suspension cells using 0.25 x 10^6^ cells/well. Unconcentrated and concentrated viral vectors from the same stocks were used to transduce pNKs on day 6 post isolation and IL-2/IL-15 cytokine-expansion. From the viral vector stocks, unconcentrated vectors (UNC) and three MOIs of 0.5; 1 and 5 concentrated viral vectors were used. Briefly, DMEM GlutaMAX with 10% (v/v) FCS and 1% (v/v) pen./strep. was added in the 24 well plate and topped with virus up to 500 µl per well. Vectofusin-1 (Miltenyi Biotec, Bergisch-Gladbach, Germany) was added at a final concentration of 10 μg/ml, the solution was mixed and incubated for 5-10 min at room temperature. pNKs were finally added to the wells, mixed and centrifuged down at 400 × g, 32°C for 90 min to increase transduction efficacy. Afterwards, the plates were set in the incubator for a 30 min rest. Finally, 500 µl of fresh NK MACS with 5% (v/v) human serum, IL-2 and IL-15 were added and the NK cells were gently resuspended by pipetting. pNK cells were maintained at a concentration of approximately 1 x 10^6^ cells/ml; medium was refreshed every 3-4 days and cells were moved to 12-well flat-bottom plates on day 7 post transduction. Transduction rates were assessed on days 3, 7, 10 and 14 post-transduction using flow cytometry to detect EGFP signal and surface CAR expression through anti-IgG1 staining (Miltenyi Biotec, Bergisch-Gladbach, Germany). Transduction efficacy was considered to be the sum of EGFP+; EGFP+CAR+ and CAR+ cells. NK cells that underwent transduction without viral vectors served as process control (PC pNKs) and cytokine-expanded NK cells (EXP pNKs) were included as negative controls.

### Lentiviral vector generation tumor target cell transduction with firefly luciferase

The lentiviral vectors encoding firefly luciferase (F.luc) were produced by transfection of HEK293-T cells using the calcium phosphate transfection kit (Thermo Fisher, Waltham, MA, USA). The packaging system assembled VSVG-pseudotyped lentiviral vectors by using the pMDLg/pRRE (Addgene #12251) plasmid encoding HIV-1 Gag and Pol and the pRSV-Rev (Addgene #12253) plasmid. Briefly, 3 x 10^6^ HEK293-T cells were seeded one day in advance in a T75 flask. In total, 30 μg total plasmid DNA (plasmids expressing transfer gene, VSV-G, Gag/Pol and Rev in a ratio of 16.2 µg: 4.5 µg: 3 µg: 6.3 µg) was mixed with 54 µl 2M calcium chloride and topped with cell culture water up to 500 µl, and 2X HEPES buffered saline was added dropwise to a final 1:1 dilution. Cell culture medium was changed from 15 ml DMEM GlutaMAX supplemented with 10% (v/v) FCS and 1% (v/v) pen./strep. to 15 ml DMEM GlutaMAX supplemented with 1.5% (v/v) FCS and 1% (v/v) pen./strep. before the transfection solution was added dropwise to the medium. Eight hours later, the medium was replaced with fresh DMEM GlutaMAX supplemented with 10% (v/v) FCS and 1% (v/v) pen./strep and the flasks were further incubated until the next day. After 24 h, cell culture medium was refreshed and the medium containing lentiviral vectors was filtered with a 0.45 µm filter and added over pre-seeded tumor target cells (UT-SCC-14, UT-SCC-42B, SCC-25 and LN-299). This procedure was repeated twice with an extended incubation time of 72 h after the third addition of lentiviral vectors. Subsequently, the transduced cells were expanded and selected with ascending puromycin (Gibco, New York, United States) concentration (1-6 µg/ml). Luciferase expression was confirmed using the Bright-Glo™ Luciferase Assay System (Promega GmbH, Walldorf, Germany) in a 1:1 dilution with 2.5x10^4^ cells/well. After a 2 min incubation time, luminescence was detected using a Centro XS^3^ LB960 Luminometer (Berthold Technologies GmbH & Co.KG, Bad Wildbach, Germany).

### Cytotoxicity luminescence assay for testing NK-effector cell functionality

To determine specific killing of the CAR-NK cells, a kinetic cytotoxicity assay was performed based on the activity of living, luciferase expressing tumor cells. Luciferase engineered tumor cells (UT-SCC-14-F.luc; UT-SCC-42B-F.luc, SCC-25-F.luc and LN-299-F.luc cells) were seeded at a concentration of 1 x 10^4^ cells/well into a 96-high binding LUMITRAC™ well plate (Greiner Bio-One GmbH, Frickenhausen, Germany) and incubated for two hours before co-culture started. Effector cells (expanded NK, CD44v6/CD19 CAR-NK, NK92 and CD44v6 CAR-NK92 cells) were added to the plates in cytokine free medium at indicated effector to target ratios (2.5:1; 1:1and 0.1:1) and incubated up to 24 h at 37°C. For the readout, D-Luciferin (Perkin Elmer, Waltham, MA) was added to the wells to a final concentration of 0.15 mg/ml and luminescence was measured using a Centro XS^3^ LB960 Luminometer (Berthold Technologies GmbH & Co.KG, Bad Wildbach, Germany) after 4, 6, 8 and 24 h of co-culture. Tumor cell viability was calculated as previously described (46): ((Emission − Background)/(Tumor cell alone − Background)) × 100%. The cytotoxic effect was determined by subtracting tumor cell viability: cytotoxicity (%) = 100% - tumor cell viability (%). Each condition was tested in triplicates and the following controls were used: tumor cells alone, medium only (background) and maximum killing (1% Triton X-100).

### Flow cytometry

CAR expression on the NK cell surface, CD44v6 and PD-L1 expression on target cells were assessed on a BD FACSCanto II, while NK cell purity and phenotyping were analyzed on a MACSQuant X. Acquired data was evaluated with the FlowJo v10.7 software (Becton Dickinson and Company, Franklin Lakes, NJ, USA). Staining was carried out according to the manufacturer’s instructions using the following antibodies: CD44v6-PE (Becton, Dickson and Company, New Jersey, USA); PD-L1-PE-Vio770; IgG1-APC; CD8-FITC; CD4-PE; CD19-PE-Vio770; CD16-PE-Vio 615; CD14-APC, CD3-VioBlue; CD45-VioGreen; NKG2A-PE; NKp44-Vio Bright B515; NKG2D-APC; NKp46-PE-Vio 615; CD16-VioGreen; CD56-APC-Vio 770; PD-1-PE-Vio770; CD69-PerCP-Vio 700; DNAM-1-Vio Bright R720; TIGIT-PE. Unless otherwise specified, all antibodies were supplied from Miltenyi Biotec, Bergisch Gladbach, Germany.

### Statistical analysis

Statistical analysis was performed with the GraphPad Prism 6 software (GraphPad, San Diego, CA, United States). Statistical significance was determined by applying the two-way ANOVA and Turkey’s multiple comparison tests; p values < 0.05 were considered significant.

## Results

### Engineering of anti-CD44v6 CAR-NK cells

**Figure 1:**
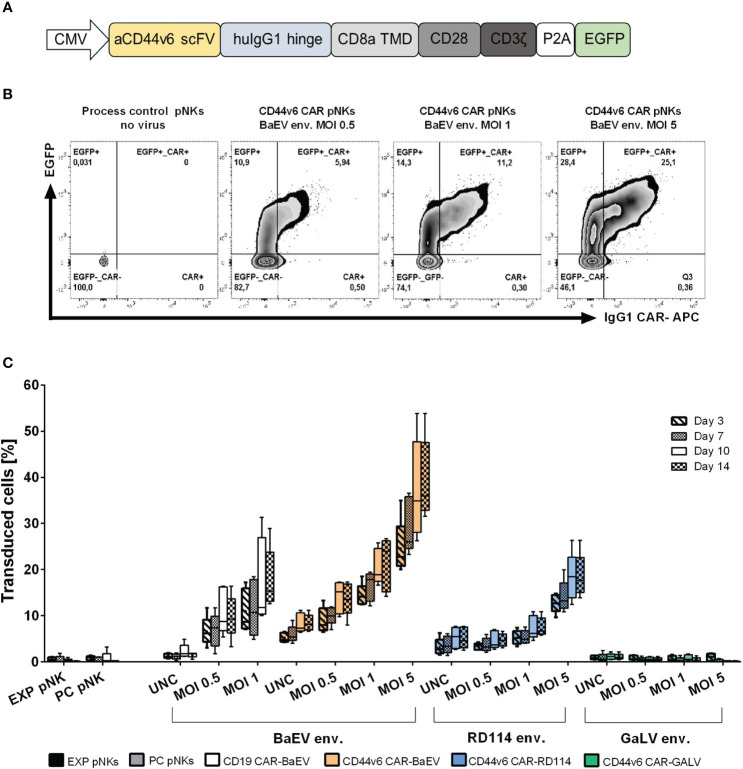
Optimization of the anti-CD44v6 retroviral vector CAR delivery and expression in primary NK cells. **(A)** Schematic representation of the anti-CD44v6 CAR construct used to generate CAR-NK cells. The anti-CD44v6 scFv (aCD44v6 scFv) derives from the BIWA8 clone of the anti-CD44v6 humanized antibody bivatuzumab. A human IgG1 hinge domain (huIgG1 hinge) links the scFv to a CD8 transmembrane domain (CD8a TMD), CD28 costimulatory domain (CD28) and CD3ζ signaling domain (CD3ζ). The genes for the enhanced GFP (EGFP) reporter and the CAR are connected through a P2A self-cleaving peptide sequence. The constitutive expression of the CAR gene in primary NK is controlled by the cytomegalovirus (CMV) promoter. The expression cassette is cloned into a gamma retroviral vector (gRV). **(B)** Example of gating strategy and comparison between different MOIs of the gamma retroviral vectors produced in HEK293-T cells and used to transduce primary NK cells. The two-parameter dot plots represent EGFP+, EGFP+CAR+ and CAR+ expression in transduced primary NKs with gRV pseudotyped with BaEV on day 10 post transduction. CAR surface expression was detected by staining with an APC IgG1 monoclonal antibody. **(C)** NK-cell transduction and percentage of total transduced cells (EGFP+, EGFP+CAR+, CAR+) at days 3, 7, 10 and 14 post transductions with gRV pseudotyped with BaEV, RD114 and GaLV. Unconcentrated viral vectors (UNC) and three MOIs of 0.5, 1 and 5 concentrated viral vectors were used to transduce NK cells. Expanded NK cells (EXP pNKs) and the process control NK cells (PC pNKs) serve as controls. Data of five independent experiments using five different healthy NK cells donors is presented as mean and standard deviation.

A second-generation CAR targeting CD44v6 was constructed using the coding sequence of the recognition domain from the humanized anti-CD44v6 antibody bivatuzumab, clone BIWA8 (43). The single chain variable fragment (scFv) targeting CD44v6 is connected through an IgG1 hinge region to the CD8a transmembrane, CD28 costimulatory and CD3ζ signaling domains. The enhanced GFP (EGFP) reporter gene was co-expressed (**Figure 1A**). The entire CAR construct is encoded by the previously described gamma retroviral vector pBullet (41). Different versions of the CAR construct were tested (**Supplementary Figure 1A**), but the decision to proceed with the CAR-P2A-EGFP construct was made to facilitate monitoring both transfection and transduction efficiency through the EGFP transgene expression (**Supplementary Figure 1B**). Transduction rates in primary NK were quantified as the total amount of EGFP+, EGFP+CAR+ and CAR+ cells as exemplified in **Figure 1B**. From the tested pseudotyped vectors, the BaEV pseudotyped gamma retroviral vectors (BaEV-gRV) induced the highest transduction rates in primary NK cells compared to the RD114 (RD114-gRV) and GaLV- pseudotyped gRV (GALV-gRV) (**Figure 1C**). By day 14 post transduction, BaEV-gRV induced mean transduction rates of 21% at an MOI of 1 for the CD44v6 CAR construct and 17.8% for a CD19 CAR construct. At the same MOI, a mean 7.6% transduction rate was obtained with RD114-gRV, while the GALV-gRV transduction was below the detection limit of 1% (**Figure 1C**). Using different MOIs of the viral vector stocks yield a titration effect in pNKs, for which the highest mean levels of CAR expression of 39.3% corresponded with the use of concentrated BaEV-gRV at an MOI of 5 (**Figure 1C**). Concentrated viral vector stocks were generated as outlined in the methods section, with additional information on vector titers available in **Supplementary Table 1**.

### Primary NK cell phenotype profile post transduction with gamma retrovirus pseudotyped with BaEV and RD114

**Figure 2:**
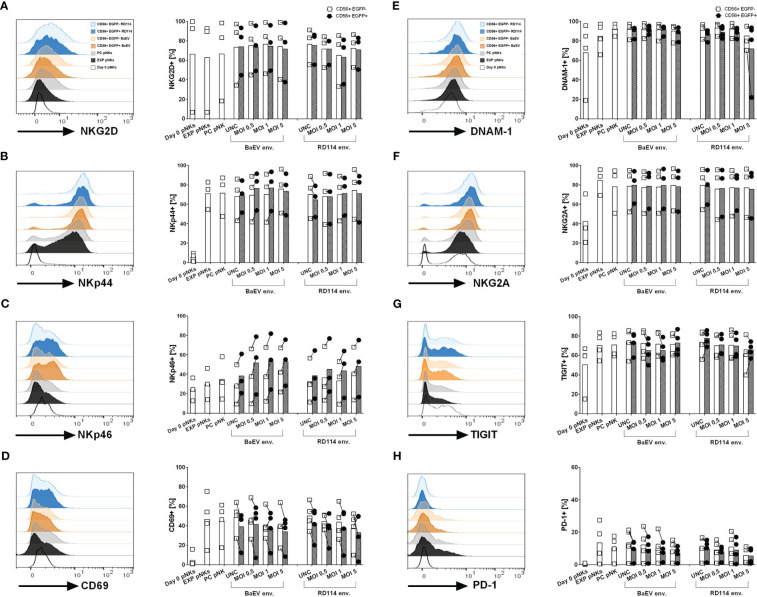
Surface marker expression on healthy donor NK and genetically modified NK cells was analyzed using flow cytometry. The histograms **(A–H)** illustrate exemplary data showcasing surface marker expression on different cell populations: unstimulated pNK cells on day 0 (Day 0 pNKs – white), expanded NK cells (EXP pNKs - black), no-vector transduced, process control NK cells (PC pNKs - grey) and viral vector transduced NK cells on day 10 post transduction. For the viral vector transduced pNKs, the following populations are highlighted: CD56+EGFP+ subpopulation from pNK cells transduced with BaEV MOI 5 (dark orange), CD56+EGFP- subpopulation from pNK cells transduced with BaEV MOI 5 (light orange), CD56+EGFP+ subpopulation from pNK cells transduced with RD114 MOI 5 (dark blue), and CD56+EGFP- subpopulation from pNK cells transduced with RD114 MOI 5 (light blue). The column dot plots display the mean and individual data of the investigated markers (n=3 NK donors for activation/inhibitory markers, n=4 NK donors for check point inhibitors). Percentages of the transduced pNK cell subpopulation (CD56+EGFP+) and untransduced subpopulation (CD56+EGFP-) expressing the activation markers NKG2D **(A)**, NKp44 **(B)**, NKp46 **(C)**, CD69 **(D)**, inhibitory markers such as DNAM-1 **(E)**, NKG2A **(F)**, as well as the check point inhibitors TIGIT **(G)** and PD-1 **(H)**.

In order to see whether transduction with different viral envelopes affects NK cell receptor expression, we checked the level of activation and inhibitory markers on the surface of donor-derived and cytokine-expanded NK cells 10 days post transduction (**Figures 2A–H**). We had a closer look at the two fractions within the transduced cells subpopulation namely untransduced CD56+ cells (CD56+EGFP-) and transduced CD56+ cell fraction (CD56+EGFP+). Due to the fact that only BaEV-gRV and RD114-gRV induced detectable levels of CD56+EGFP+ cells, surface marker profiles are presented only in these versions of genetically engineered NK cells. While the NKG2D and DNAM-1 activation markers were present on NK cells at similar levels on the isolation day and 10 days post cytokine-stimulation (**Figures 2A, E**), other activation markers like NKp44, NKp46 and CD69 were upregulated during cytokine-expansion of NK cells (**Figures 2B, C, D**). The inhibitory marker NKG2A was found at higher levels in NK cells that were cytokine stimulated, with similar expression in all transduced cells (**Figure 2F**). TIGIT and PD-1 immune checkpoint inhibitor expression was also upregulated on NK cells after cytokine expansion (**Figures 2G, H**). No difference in the expression of investigated surface markers was detected between the BaEV and RD114 viral envelope proteins. The activation marker NKp46 was upregulated in the CD56+EGFP+ cell fraction as compared to the CD56+EGFP- fraction. In two donors, higher PD-1 expression was found in expanded NK cells, process control NK cells, as well as in the untransduced, CD56+EGFP- cells, compared to CD56+EGFP+ cells (**Supplementary Figure 2A**).

### Anti-CD44v6 CAR-NK cells display redirected killing activity against HNSCC cell lines compared to controls

**Figure 3:**
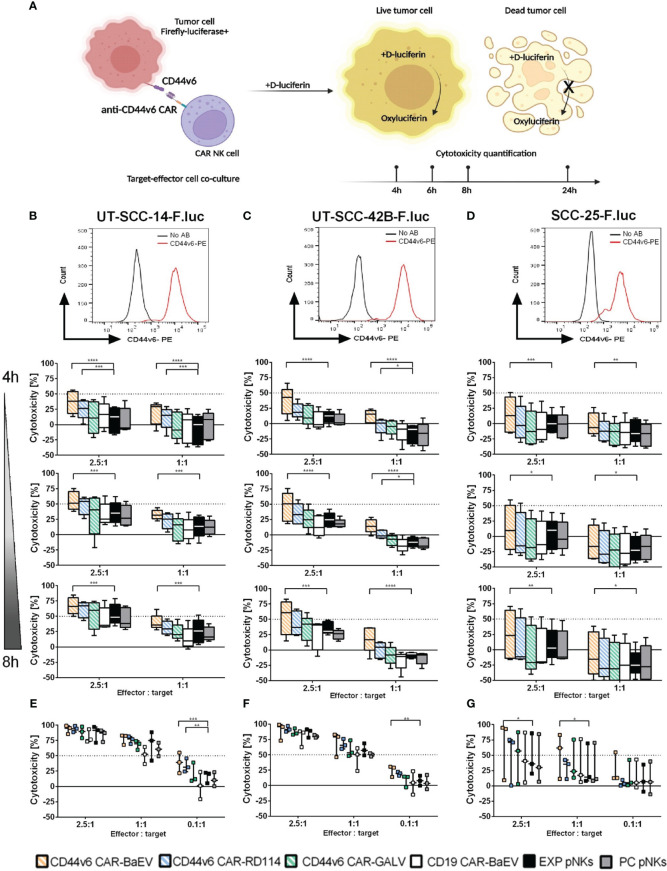
Killing efficacy of anti-CD44v6 CAR-NK cells against different HNSCC cell lines after 4, 6, 8 and 24 h of co-culture. **(A)** Schematic representation of the kinetic killing assay in which target cells genetically modified to express firefly luciferase (F.luc) are co-cultured with effector NK cells. Killing efficacy is monitored by recording living cells through the use of D-luciferin as substrate. This substrate is reduced to oxyluciferin by living cells. The bioluminescent signal is quantified and normalized to indicate cell killing. **(B–D)** CD44v6 positive UT-SCC-14-F.luc; UT-SCC-42B-F.luc and SCC-25-F.luc cells are set in co-culture with EXP pNKs; PC pNKs and CD44v6 CAR-NKs at 2.5:1 and 1:1 effector to target ratios. Killing efficacy is quantified after 4, 6 and 8 h of co-culture (n=5 donors). **(E–G)** Killing efficacy against the same target cell lines was checked after 24 h using 2.5:1, 1:1 and 0.1:1 effector to target ratios (n=3 donors). Data is presented as mean and standard deviation of 5 or 3 independent experiments. Descriptive statistics were calculated using two-way ANOVA and Turkey’s multiple comparison test (* p<0.05; **p<0.01; ***p<0.001; ****p<0.0001).

Functionality of genetically modified NK cells was determined through co-cultures with firefly-luciferase (F.luc) expressing target cells and quantified at different time points by measuring the activity of living target cells that reduced the D-luciferin substrate to oxyluciferin as shown in **Figure 3A**. Using this setup, killing kinetics was measured in the same well at different time points. For this assay, F.luc positive HNSCC cell lines UT-SCC-14-F.luc, UT-SCC-42B-F.luc and SCC-25-F.luc were analyzed for their CD44v6 surface expression (**Figures 3B–D**) and NK cells transduced with an MOI of 1 from each of the CAR variants were chosen as effector cells. Transgene expression level was in average between 21% for BaEV-gRV, 7.6% for RD114-gRV and below 1% for GALV-gRV. An anti-CD19 CAR (CD19 CAR-BaEV) as well as expanded NK cells (EXP pNKs) and a no-virus process control pNKs (PC pNKs) were used as controls. All effector cells were tested functionally on day 17 post isolation and cytokine expansion using the luminescence-based cytotoxicity assay. In the case of the UT-SCC-14-F.luc target cells, CD44v6 CAR-NKs transduced with BaEV-gRV induced significantly higher killing than cytokine-expanded NK cells at effector to target ratios of 2.5 to 1 and 1 to 1 even at the early time points of 4, 6 and 8 h of the co-culture (**Figure 3B**). After 24 h, a significant CAR effect was seen only at the lowest 0.1 to 1 effector to target ratio against the same cell line (**Figure 3E**). Similar effects were visible in the case of the UT-SCC-42B-F.luc cells, but a significant difference between the CD44v6 CAR-BaEV and the expanded NK cells control was recorded starting with 6 h post co-culture setup (**Figures 3C, F**). For the SCC-25-F.luc cell line, a higher donor variation in killing efficacy was seen (**Figure 3D**). Significant difference between the CD44v6 CAR-BaEV and the expanded NK cells control in killing SCC-25-F.luc targets was visible after 6 h of co-culture and remained consistent for 24 h (**Figure 3G**). Overall, anti-CD44v6 CAR-NK cells outperformed cytokine-expanded primary NKs from the same donor with an approximately two- to threefold increase in killing efficacy. We recorded a different killing kinetics for two donors found to have high PD-1 expression (**Supplementary Figure 2A**). PD-1/PD-L1 inhibition is an established treatment option for HNSCC patients (47). The tested cell lines were also found to express PD-L1 at different levels (**Supplementary Figure 2B**). NK cells from donors expressing PD-1 exhibited poorer performance against UT-SCC-42B and SCC-25 HNSCC cell lines during the functionality test (**Supplementary Figure 2C**). A direct correlation between PD-1 expression and the cytotoxicity of effector NK cells was indicated by the difference between CAR-NK and expanded NK cell cytotoxicity. As PD-1 expression increased, the discrepancy in killing efficacy decreased (**Supplementary Figures 2D, E**).

To determine if the anti-CD44v6 CAR-NK cells also target other types of tumor cells, genetically modified NK cells were set in co-culture with a glioblastoma cell line, LN-299-F.luc. Although LN-299-F.luc cells express CD44v6 to a lesser extent than HNSCC cell lines (**Supplementary Figure 3A**), specific CAR mediated killing was induced at an effector to target ratio of 1 to 1 after 4 h (**Supplementary Figure 3B**) which was maintained up to 8 h of co-culture (**Supplementary Figures 3C, D**).

We also conducted efficacy testing comparing primary NK cell-derived CARs with CAR-NK-92 cells. Anti-CD44v6 CAR-NK-92 cells were generated from the NK-92 cell line using a BaEV-gRV with a co-expressed EGFP reporter. Transduction efficacy in NK-92 cells was still over 93% after long-term culture (**Supplementary Figure 4A**), while positively transduced primary NK cells at an MOI of 1 had an average CAR expression of 21% (**Supplementary Figure 4B**). We compared CAR-mediated cytotoxicity using transduced primary NKs and transduced NK-92 cells after 4, 6 and 8 h of co-culture with target HNSCC cell lines (**Supplementary Figures 4C, D, E**). Killing efficacy of the tested CARs was target cell line depended, with higher killing seen in UT-SCC-14-F.luc and UT-SCC-42B-F.luc targets. For these two target cell lines, significant difference between primary NKs and NK-92 was detected starting at 4 h post co-culture, and remained constant throughout the 8 h of experiment (**Supplementary Figures 4C, D**). The SCC-25 cell line, was effectively targeted and killed by both CAR-NK cells at similar rates throughout the assay (**Supplementary Figure 4E**). Despite their high CAR expression, the difference in efficacy of CD44v6 CAR-NK92 compared to the NK-92 control was marginal. An advantage of NK cell redirection with CAR molecules was detected only at later time points, and seemed to be more pronounced against the SCC-25 target cell line (**Supplementary Figures 4C, D, E**).

## Discussion

Effective treatment of HNSCC still remains challenging despite current advancements in immunotherapy. Approved immunotherapies like PD-1 checkpoint inhibitors have limited efficacy, extending overall survival with around 13 months in combination with chemotherapy (48). Despite intense pre-clinical efforts, the success of CAR-T cell therapy has yet to be transferred for solid tumor treatment (49). In this setting, natural killer cells provide a good alternative to the T effector-cell. Through the use of CARs, NK cells are redirected towards specific surface antigens on tumor cells, allowing for antigen-specific targeting and enhanced killing (50). In this study, we genetically modified NK cells to express CARs redirected against the CD44v6 surface protein, a target associated with HNSCC during metastasis and disease progression (51, 52). Various CD44v6 expression levels were previously reported in HNSCC, depending on the choice of antibodies, detection method and scoring systems used. Overall, CD44v6 was found to be higher expressed than other surface markers in HNSCC, underlining its use as a good option for solid tumor targeted therapy (53). Prior research demonstrated the usefulness of CD44v6 as a solid tumor target in both CAR-T and CAR-NK preclinical applications (29, 38, 40), as well as in phase I/II clinical trials (clinical trials.gov NCT04097301 and NCT04427449).

In the presented study, a second-generation anti-CD44v6 CAR was successfully expressed in primary NK cells when using a pseudotyped gRV vector. Safety concerns in regard to insertional oncogenesis limited gRV use in gene therapy applications despite their efficiency in genetic modification of cells (54). However, vector optimization (55) enabled gRV use in approved cell therapies such as the anti-CD19 CAR-T Yescarta^®^ (56). Furthermore, gRV were successfully and safely tested in a clinical trial using genetically modified NK cells (17). Efficient genetic modification of primary NK cells relies on the utilization of an optimal envelope for vector pseudotyping. In this paper we describe the comparative use of three different envelopes for NK cell transduction: BaEV, RD114 and GALV. Alpha retroviral vectors pseudotyped with RD114 were previously reported to induce higher transduction rates in blood-derived primary NK cells compared to gamma retroviral and lentiviral vectors when using CAR constructs (57). Transduction rates of up to 87.4% were also achieved by utilizing RD114-pseudotyped gRV to express a CD19-CAR into cord-blood derived NK cells (11). In contrast to published data, we obtained mean transduction rates of 7.6% in blood derived primary NKs when using RD114-gRV, and rates below detection limit for GALV-gRV modified NK cells. We achieved the highest transduction rates when using BaEV-gRV, which is in line with prior studies by Colamartino et al. (58) and Bari et al. (59). These studies demonstrated a transduction efficacy of over 38% in primary NK cells when using lentiviral vectors pseudotyped with BaEV at MOIs of 5, as opposed to the RD114 and vesicular stomatitis virus G protein (VSV-G) pseudotyped variants.

For the present study, the design of the CAR molecule was based on a T cell specific construct (42), with intracellular signaling domains that, although not NK cell specific, were previously shown to induce efficient and targeted killing for an anti-CD19 CAR-NK cell therapy; and consequently with notable therapeutic outcome in a phase I clinical trial (17, 60).

Examination of NK cell phenotype post transduction revealed an increase in the expression of markers such as NKp44, NKp46, CD69 and NKG2A post cytokine-expansion of NK cells. No correlation with the used viral envelope proteins and NK cell surface marker expression was found. Similar NK cell phenotypes were reported when using either RD114 on alpha retroviral (57), BaEV on lentiviral (58) or RD114 on gamma retroviral vectors (11). We noticed an upregulation of NKp46 expression in the transduced CD56+EGFP+ cell subpopulation, while other studies reported a slightly higher NKp44 positive population in transduced cells as compared to expanded cells (11). The differences in receptor expression observed in expanded and transduced NK cells could be attributed to the source of NK cells (peripheral blood versus cord blood) or to the expansion methods used (cytokine-expansion versus feeder-cell expansion).

The efficacy of NK cells in HNSCC was and is being investigated in various clinical trials, that include the use of off-the-shelf NK products combined with monoclonal antibodies (clinical trials.gov NCT05674526), autologous and *ex vivo* expanded NK cells (clinical trials.gov NCT00717184) with or without bispecific antibodies (clinical trials.gov NCT05099549) as well as genetically modified NK cells expressing anti-PD-L1 (clinical trials.gov NCT04847466) or anti-NKG2D-ligand CARs (clinical trials.gov NCT03415100). In our study, we demonstrate two- to threefold enhanced cytotoxicity against HNSCC *in vitro* when using CD44v6-targeted CAR-NK cells, as compared to expanded non-engineered NK cells. Similar results were obtained with anti-CD44v6 CAR-NK cells directed against triple negative breast cancer (40) and anti-CD44v6 CAR-T cells targeting HNSCC (29), or lung and ovarian carcinomas (38). Using anti-CD44v6 CAR-NK cells, we also saw effective killing against the glioblastoma cell line LN-299 expressing low levels of CD44v6. Currently, an anti-HER2-CAR-NK-92 cell-based therapy is under investigation in a phase I/II clinical trial (clinical trials.gov NCT03383978) for glioblastoma. However, additional CAR targets are likely beneficial for the treatment of such a challenging tumor. The CD44v6 splice variant is expressed also by brain tumor stem-like cells (BTSC), implying a promising target for redirected therapy of brain tumors (61). These findings highlight CD44v6 as a promising target for CAR therapy across multiple cancer types.

Inter-donor variability in primary NK cells can significantly influence NK cell phenotype, inherent transduction efficacy and overall function (62). Prior studies revealed the occurrence of functionally different capabilities in eliminating tumor cells between different NK cell subtypes and individuals (63, 64). In accordance to these findings, we have seen donor variability in transduction efficacy and basal functionality of expanded NK cells, as well as donor-to-donor variation in NK cell phenotypes post cytokine-expansion. PD-1 expression on primary NK cells was reported in different types of cancer (65–67) and linked to poor anti-tumor activity (68). We found PD-1 expression in two of our tested donors in cytokine-expanded NK cells and the CD56+EGFP- subpopulation of transduced cells. Both the use of IL-2 and IL-15 as well as a combination of IL-12, IL-15, and IL-18 may induce PD-1 expression on expanding NK cells (67, 69). We noticed that PD-1 expression in the CD56+EGFP- subpopulation of CAR-NK cells can affect their overall cytotoxicity, suggesting that the presence of this immune check point could be a limiting factor for CAR-therapy (as shown in **Supplementary Figure 2**). Nevertheless, further PD-1 blocking experiments are required to determine the validity of the effect of PD-1 presence on NK cells. In support of this, recent preclinical research has highlighted the advantages of incorporating competitive PD-1 blocking by co-expression alongside the CAR construct (40). Moreover, a phase II clinical trial evaluating anti-PD-L1 CAR-NK cells together with an immune check point inhibitor for the treatment of advanced HNSCC is currently recruiting (clinical trials.gov NCT04847466).

In terms of effector cells, NK-92 cells were reported to have higher cytotoxicity compared to donor-derived NK cells due to increased granzyme content and fewer inhibitory killer Ig-like receptors (KIR) receptors (70). In contrast, we recorded a superior cytotoxic effect of the expanded primary NK and anti-CD44v6 CAR-NK compared to the NK-92 or anti-CD44v6 CAR-NK-92 cell lines, despite a stable, over 95% CAR expression in the last-mentioned effector cell type. Furthermore, there seemed to be a marginal effect of the CAR presence on NK-92 cells. These observations may be explained by the different expansion and activation methods applied to the two cell types. The IL-2/IL-15 cytokine combination used for primary NK cell culture might have induced stronger signaling in these cells through the IL-2/IL-15 receptor complex, potentially enhancing their killing efficacy compared to the IL-2 stimulated NK-92 cells (71). Furthermore, there seemed to be a tumor cell line susceptibility to effector cell killing, with UT-SCC-14-F.luc and UT-SCC-42B-F.luc cell lines being more efficiently killed by primary NK cells as opposed to NK-92 cells, likely as a result of a more favorable match between primary NK cell receptors and tumor target ligands (72).

While our findings show that anti-CD44v6 CAR-NK cell therapy has specific anti-tumor cell line efficacy, further testing is required to optimize the efficacy of this cell-based therapy against HNSCC. A more comprehensive examination using patient derived tumor material or 3D tumor models would underline the potential of this CAR-NK cell therapy under the appropriate microenvironment. Furthermore, *in vivo* preclinical testing would aid in understanding CAR-NK cell longevity and homing patterns to various body compartments, as well as determining whether it induces off-tumor effects.

## Data availability statement

The raw data supporting the conclusions of this article will be made available by the authors, without undue reservation.

## Ethics statement

The studies involving humans were approved by the State Chamber of Physicians of Saxony, Germany. The studies were conducted in accordance with the local legislation and institutional requirements. Written informed consent for participation was not required from the participants or the participants’ legal guardians/next of kin because blood donors are anonymous and volunteers.

## Author contributions

ISC: Conceptualization, Formal Analysis, Investigation, Methodology, Visualization, Writing – original draft, Writing – review & editing, Project administration. JF: Investigation, Methodology, Writing – review & editing, Visualization. AQ: Visualization, Writing – original draft, Formal Analysis. CB: Investigation, Methodology, Writing – review & editing. HA: Resources, Writing – review & editing. UST: Methodology, Resources, Writing – review & editing. SF: Resources, Writing – review & editing, Conceptualization. UK: Conceptualization, Funding acquisition, Project administration, Supervision, Writing – review & editing. DS: Conceptualization, Formal Analysis, Methodology, Project administration, Supervision, Visualization, Writing – original draft, Writing – review & editing. TG: Conceptualization, Formal Analysis, Methodology, Resources, Supervision, Visualization, Writing – original draft, Writing – review & editing, Project administration, Funding acquisition.
