# Supplementary material for: CD44v6 specific CAR-NK cells for targeted immunotherapy of head and neck squamous cell carcinoma
Source: Front Immunol. 2023 Nov 10;14:1290488. doi: 10.3389/fimmu.2023.1290488 (PMC10667728; doi:10.3389/fimmu.2023.1290488)
Supplement: Supplementary file 1 [file DataSheet_1.docx]

Supplementary Material

CD44v6 specific CAR-NK cells as targeted immunotherapy for head and neck squamous cell carcinoma

Ioana Sonya Ciulean^1*^; Joe Fischer^1^; Andrea Quaiser^1^; Christoph Bach^1^; Hinrich Abken^2^; Uta Sandy Tretbar^1^; Stephan Fricke^1^; Ulrike Koehl^1, 3^; Dominik Schmiedel^1, 3†^; Thomas Grunwald^1†*^

*** Correspondence:** Ioana Sonya Ciulean [sonya.ciulean@izi.fraunhofer.de](mailto:sonya.ciulean@izi.fraunhofer.de) ;Thomas Grunwald [thomas.grunwald@izi.fraunhofer.de](mailto:thomas.grunwald@izi.fraunhofer.de)

##
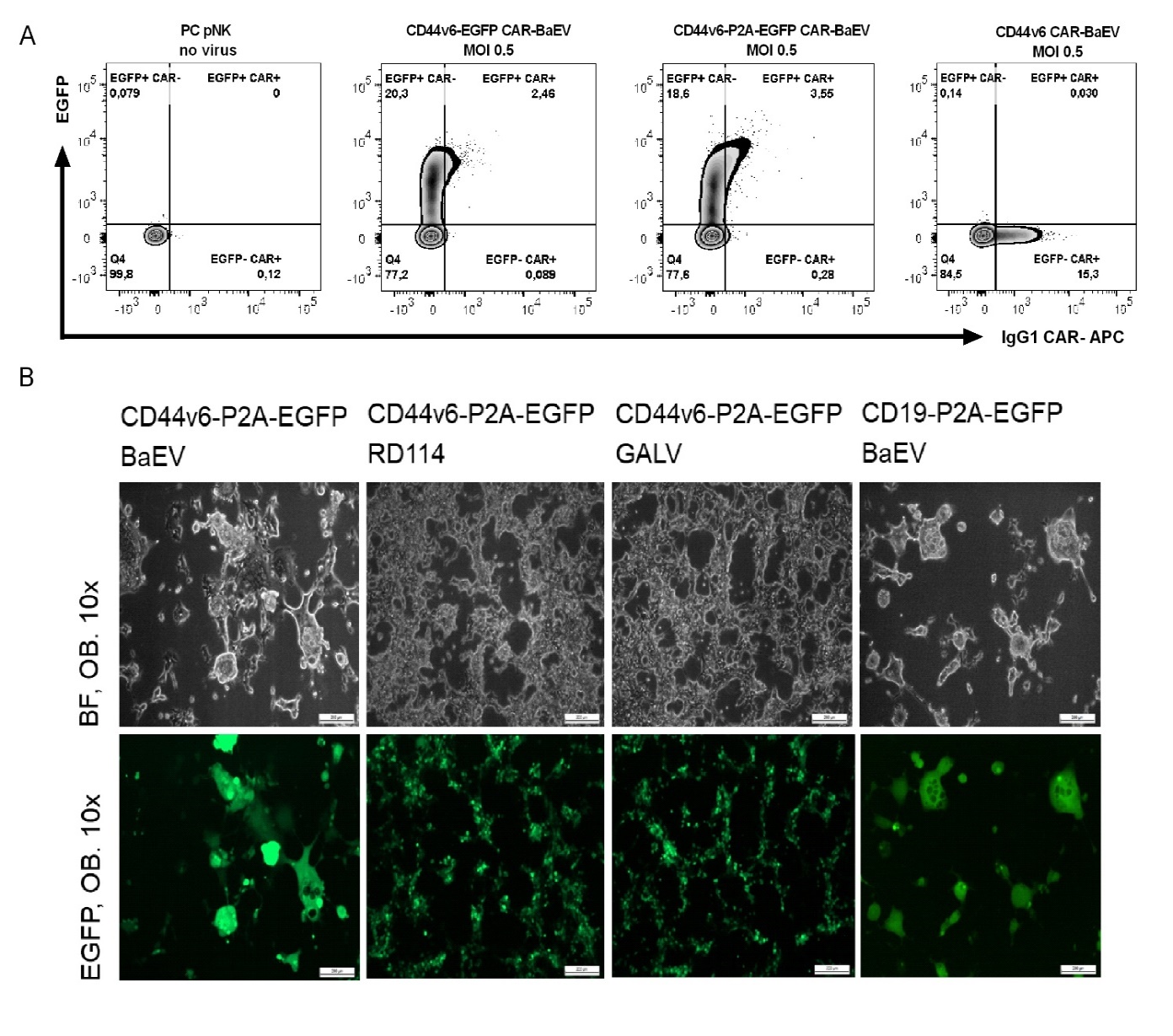
Supplementary Figures

**Supplementary Figure 1.** CD44v6 CAR construct expression in primary NK cells and viral vector stock production for primary NK transduction. **(A)** Three CD44v6 CAR constructs were evaluated for their expression in primary NK cells. The two-parameter dot plots present data from the process control primary NK cells (PC pNKs), mock transduced without virus; and primary NK cells transduced with a fused EGFP to the CD44v6 CAR construct (CD44v6-EGFP), a version including a self-cleavable P2A site that links the CAR to EGFP (CD44v6-P2A-EGFP) and a CD44v6 CAR without EGFP (CD44v6 CAR). Baboon envelope pseudo-typed gamma retroviral vectors (BaEV-gRV) were used to transduce NK cells on day 6 post isolation. CAR and EGFP expression were determined 11 days post transduction by flow cytometry after staining with an anti- IgG1 monoclonal antibody. **(B)** HEK293-T producer cells were used for the generation of the three gRV pseudotypes: BaEV-gRV, RD114-gRV and GALV-gRV. Transfection efficacy was checked under the microscope before vector collection and was considered successful by the presence of EGFP+ cells and the cytopathic effect in the case of BaEV (bright field, OB.10x and EGFP filter, OB.10x).


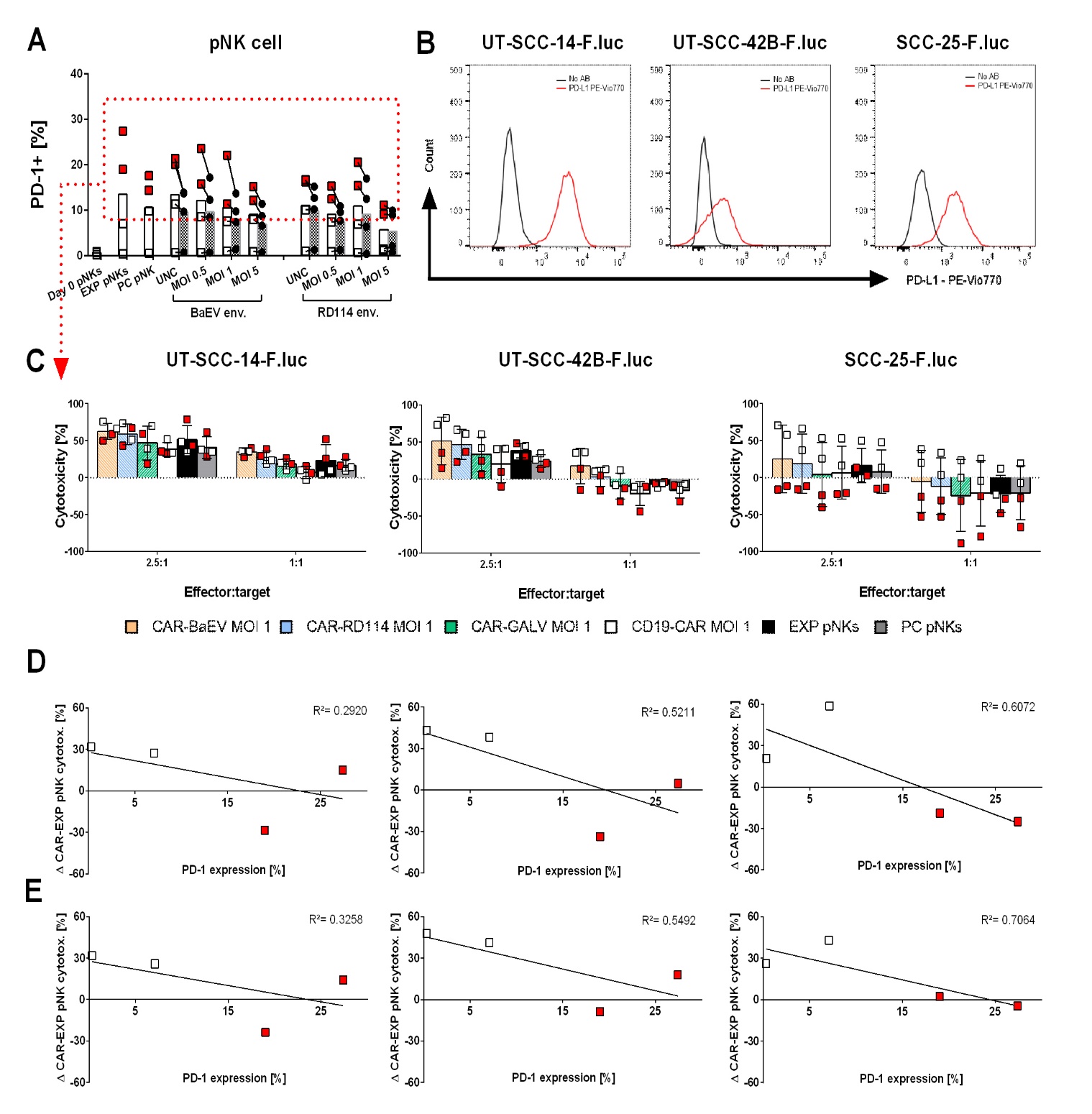


**Supplementary Figure 2**. PD-1 expression in primary NK cells affects killing efficacy of PD-L1 expressing target cells. **(A)** Two NK cell donors were found to express high PD-1 in cytokine-expanded primary NKs (EXP pNKs, PC pNKs and the CD56+EGFP- subpopulation of transduced NK cells). **(B)** Surface PD-1 ligand expression (PD-L1) was checked in HNSCC target cell lines. **(C)** Killing efficacy at 8 h post co-culture with HNSCC cell lines. The data includes individual killing percentages of each donor along with the group mean and standard deviation. Difference between CAR and EXP pNK cytotoxicity of individual donors presented as linear regression, 8 hours post co-culture and provided for two effector-to target ratios 1:1 **(D)** and 2.5:1 **(E)**. Red squares represent donors B and E, who exhibit high PD-1 expression.


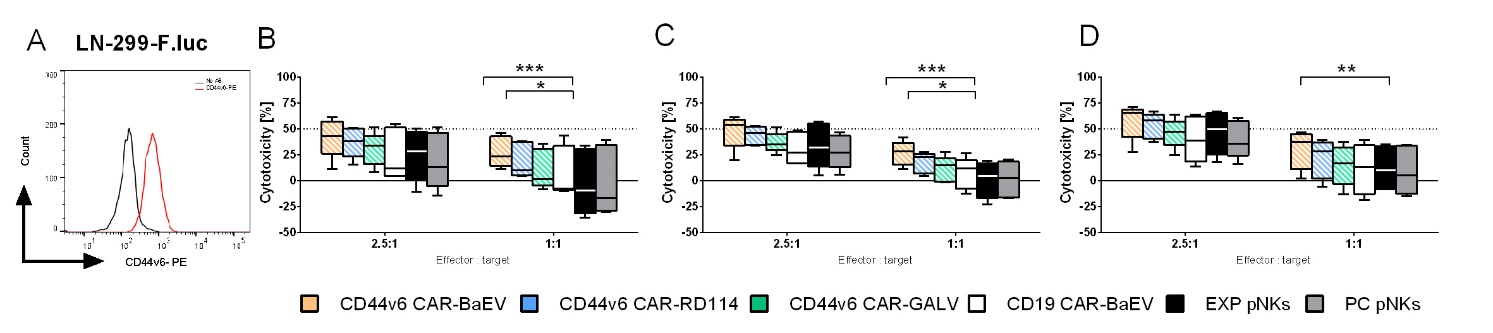


**Supplementary Figure 3.** Killing efficacy kinetics of anti-CD44v6 CAR-NK cells against CD44v6-low expressing glioblastoma LN-299-F.luc cell line. **(A)** Expression level of CD44v6 in LN-299-F.luc cells detected using an anti-CD44v6 antibody. LN-299-F.luc cells are set in co-culture with EXP pNKs, PC pNKs and anti-CD44v6 CAR-NK cells at 2.5 :1 and 1:1 effector to target ratio. Killing efficacy is quantified after 4 **(B)**, 6 **(C)** and 8 h **(D)** of co-culture (n=5 different NK cell donors). Data of five independent experiments is presented as mean and standard deviation. Descriptive statistics were calculated using two-way ANOVA and Turkey’s multiple comparison.

**
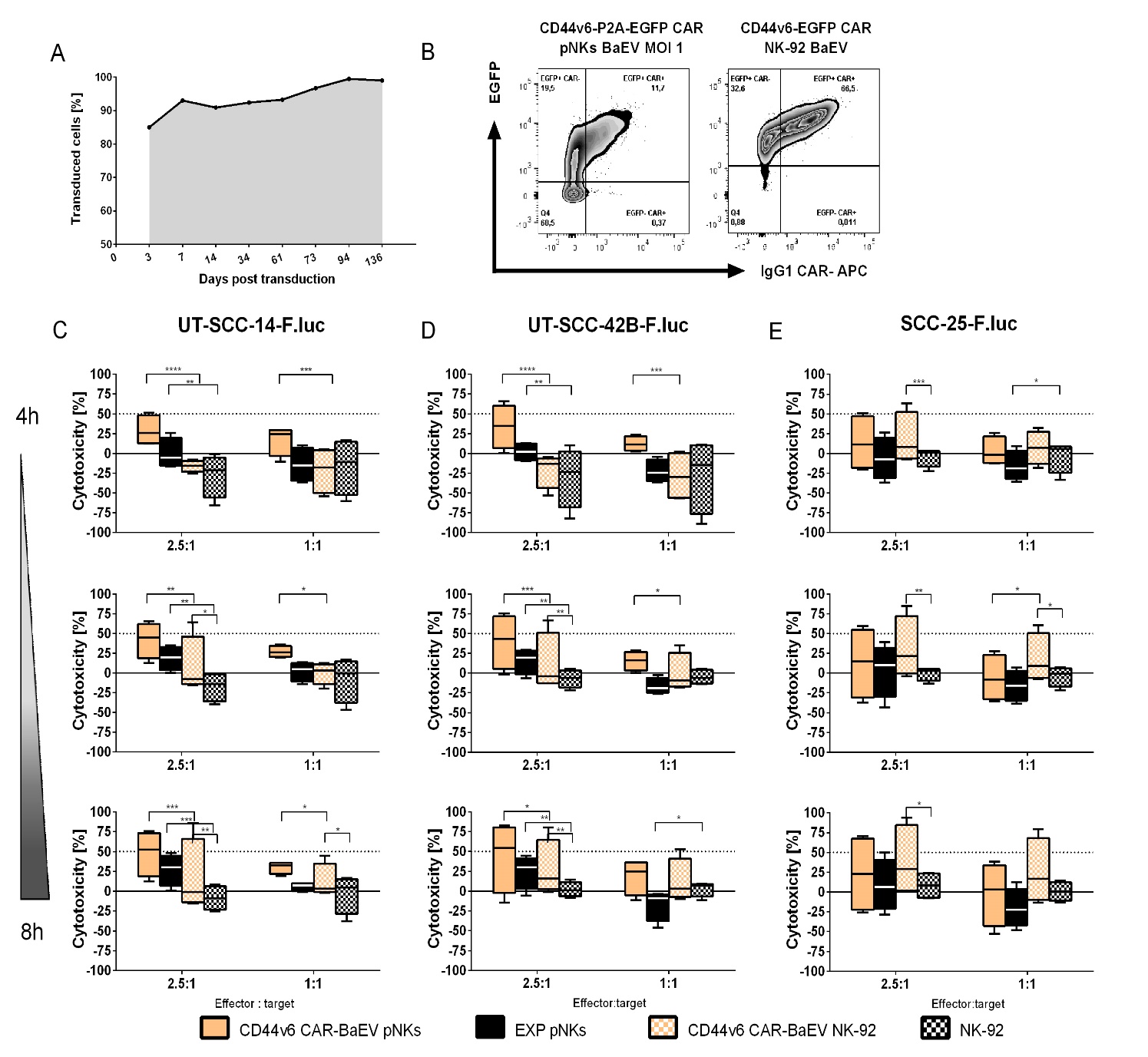
**

**Supplementary Figure 4**. Killing efficacy comparison between primary anti-CD44v6 CAR-NKs (n=4 donors) and an anti-CD44v6 CAR-NK-92 cell line. **(A)** The linear graph represents transduction efficacy of the anti-CD44v6 CAR in NK-92 cells in long-term culture. Data is presented as sum of EGFP+, EGFP+CAR+ and CAR+ percentages expressed by transduced NK-92 cells. **(B)** Example of comparison EGFP and CAR expression in primary NK and NK-92 cells. Baboon envelope pseudo-type gamma retroviral vectors were used to transduce both cell types. The CAR construct used for NK-92 transduction had a fused EGFP reporter gene, while a P2A-EGFP version was used for primary NK cell transduction. Effector NK cells were co-cultured with target UT-SCC-14-F.luc (C), UT-SCC-42B-F.luc (D) and SCC-25-F.luc (E) HNSCC cell lines. Killing efficacy was determined after 4, 6 and 8 h of co-culture. Data is presented as mean and standard deviation of four independent experiments used to summarize the observations. The descriptive statistics were calculated using two-way ANOVA and Turkey’s multiple comparison test (* p<0.05; ** p<0.01; *** p<0.001; **** p<0.0001).

## Supplementary Tables

**Supplementary Table 1**. Titer of gamma retroviral vector stocks after concentration

| **gRV + indicated**  **envelope expression plasmid** | **Concentrated viral vector**  **titer in**  **transducing units per ml (TU/ml)** |
| --- | --- |
| CD44v6-P2A-GFP + BaEV | 6.98 x 10^6^ |
| CD44v6-P2A-GFP + RD114 | 1.55 x 10^7^ |
| CD44v6-P2A-GFP + GALV | 1.68 x 10^7^ |
| CD19 -P2A-GFP + BaEV | 1.46 x 10^6^ |
